# MEMS-Based Electrochemical Seismometer Relying on a CAC Integrated Three-Electrode Structure

**DOI:** 10.3390/s21030809

**Published:** 2021-01-26

**Authors:** Xu She, Junbo Wang, Deyong Chen, Jian Chen, Chao Xu, Wenjie Qi, Bowen Liu, Tian Liang

**Affiliations:** 1 State Key Laboratory of Transducer Technology, Aerospace Information Research Institute, Chinese Academy of Sciences, Beijing 100190, China; shexu18@mails.ucas.edu.cn (X.S.); dychen@mail.ie.ac.cn (D.C.); chenjian@mail.ie.ac.cn (J.C.); xuchao16@mails.ucas.edu.cn (C.X.); qiwenjie16@mails.ucas.edu.cn (W.Q.); liubowen17@mails.ucas.edu.cn (B.L.); liangtian18@mails.ucas.edu.cn (T.L.); 2 School of Electronic, Electrical and Communication Engineering, University of Chinese Academy of Sciences, Beijing 100049, China

**Keywords:** electrochemical seismometer, MEMS, integrated three-electrode structure, sensitivity, noise level

## Abstract

This study developed a MEMS-based electrochemical seismometer relying on a cathode–anode–cathode (CAC) integrated three-electrode structure where two cathodes were positioned on two surfaces of a silicon wafer, while one anode was positioned on the sidewalls of the through holes of the silicon wafer. Device design and numerical simulations were conducted to model the functionality of the three-electrode structure in detecting vibration signals with the key geometrical parameters optimized. The CAC integrated three-electrode structure was then manufactured by microfabrication, which demonstrated a simplified fabrication process in comparison with conventional four-electrode structures. Device characterization shows that the sensitivity of the CAC microseismometer was an order of magnitude higher than that of the CME6011 (a commercially available four-electrode electrochemical seismometer), while the noise level was comparable. Furthermore, in response to random vibrations, a high correlation coefficient between the CAC and the CME6011 (0.985) was located, validating the performance of the developed seismometer. Thus, the developed electrochemical microseismometer based on an integrated three-electrode structure may provide a new perspective in seismic observations and resource explorations.

## 1. Introduction

A seismometer is essentially a sensor that detects vibration signals. At present, its core application scenarios include seismic observations, resource explorations, and planetary research. These all have high requirements for the low-frequency performances of seismometers [1,2,3,4,5]. However, conventional seismometers based on moving-coils, optic-fibers, and piezoelectrics cannot effectively address these requirements due to poor low-frequency characteristics [6,7,8,9,10,11,12,13]. Meanwhile, electrochemical seismometers that have appeared in recent years use electrolytes as inertial masses and are featured with large working angles, high sensitivities, low noise levels, and excellent low-frequency performances. Therefore, they can, to an extent, meet the actual needs in seismic observations and resource explorations [14,15].

In conventional electrochemical seismometers, sensitive electrodes were fabricated with woven metallic meshes and they suffered from the problems of low production efficiency and poor consistency [16]. In order to address these issues, He et al. and Deng et al. fabricated sensitive electrodes based on microfabrication. Although high-consistency sensitive electrodes were manufactured, multiple wafers were required and they cannot be properly aligned [17,18,19].

In 2013, Huang et al. developed integrated sensitive electrodes where only one single wafer was used, which, however, had a key limitation of effective electrode areas, resulting in compromised device sensitivities [20]. In 2016, Sun et al. put forward MEMS-based single-wafer electrochemical seismometers relying on an SU-8 insulating layer, which was prone to damage due to the design of floating electrodes [21]. In 2019, Zheng et al. developed an integrated structure where both cathodes and anodes were formed on the same side of the silicon wafer, which, however, suffered from complex fabrication steps (e.g., seven photolithography steps) due to the four-electrode (anode–cathode–cathode–anode, ACCA) structure [22].

In order to deal with the aforementioned challenges, in this study, a MEMS-based electrochemical seismometer was developed where a cathode–anode–cathode (CAC) integrated three-electrode structure was utilized. Due to the removal of one anode, corresponding fabrication processes can be simplified while device performances can be maintained in comparison with the four-electrode structure. More specifically, in this study, both theoretical analysis and numerical simulations were conducted to model the three-electrode structure, followed by electrode fabrication and performance characterization, which is described in the following sections.

## 2. Structure and Simulation

### 2.1. Device Structure

**Figure 1:**
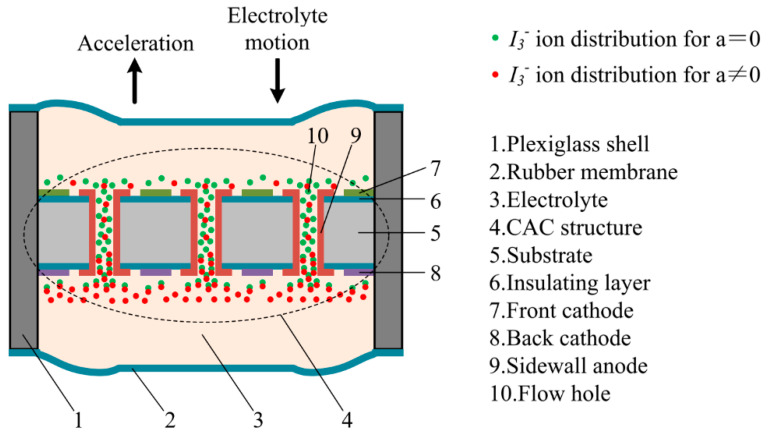
Schematic of the MEMS-based electrochemical seismometer relying on a cathode–anode–cathode (CAC) integrated three-electrode structure (e.g., 7—front cathode, 8—back cathode, and 9—sidewall anode). Variations in environmental vibrations lead to movements of 2—rubber membrane and 3—electrolyte, resulting in ion distribution imbalances on the CAC electrodes and thus producing current outputs.

The sensitive components of the MEMS electrochemical seismometer are composed of four main parts, which are the CAC integrated three-electrode structure (“7” front cathode, “8” back cathode, and “9” sidewall anode), an electrolyte, two rubber membranes, and a plexiglass shell (see Figure 1). In comparison with the four-electrode structure of the ACCA where both anodes are applied with the same electrical potential without current signal outputs, in this study, two anodes were connected together to form the three-electrode structure of the CAC, which can simplify the electrode structure without compromises in electrode performances. In order to form the CAC structure, in this study, two cathodes were positioned on two sides of the silicon wafer for the consideration of symmetry, while the only anode was positioned on the sidewalls of the through-holes of the silicon wafer.

As to the working mechanism, the electrolyte is a mixed solution of elemental iodine and potassium iodide. They will undergo a complex reaction in the solution: $I_{2}+I^{-}↔I_{3}^{-}$. A DC voltage of 0.3 V is applied to the anode and cathode and a reversible oxidation–reduction reaction occurs on the surface of the cathode and anode: the cathode is $I_{3}^{-}+2e^{-}\to 3I^{-}$, and the anode is $3I^{-}-2e^{-}\to I_{3}^{-}$. Variations in environmental vibrations lead to the movements of “2”—rubber membrane and the relative displacement of “3”—electrolyte as the liquid mass compared with CAC electrodes. Thus, the static balances between ion distributions and electrochemical reactions on the electrode surfaces are broken, leading to differential current outputs on the cathodes of the CAC structure (see Figure 1).

According to the working principle of the MEMS-based electrochemical seismometer, it can be decomposed into two modules for analysis. One is the mechanical module, which is responsible for detecting external vibration and converting the vibration speed into the relative movement speed of the electrolyte and the electrode. The second is the electrochemical module, which is responsible for detecting changes in the ion concentration gradient in the electrolyte and converting this change into a current signal.

The mechanical module selects the speed of the electrolyte as the research object and studies the conversion process from the external vibration speed $v_{e}$ to the flow speed $v_{i}$ of the electrolyte in the channel. The transfer function of the mechanical module is:

(1) $$\left|W_{1}\left(\omega \right)\right|=\left|\frac{v_{i}\left(\omega \right)}{v_{e}\left(\omega \right)}\right|=\frac{\rho L\omega^{2}}{\sqrt{\rho^{2}L^{2}\left(\omega^{2}-\omega_{0}^{2}\right)+R_{h}^{2}\omega^{2}S_{ch}^{2}}}$$

where ρ is the density of the electrolyte, L is the length of the channel, $S_{ch}$ is the cross-sectional area of the channel, $\omega_{0}$ is the natural frequency, and $R_{h}$ is the channel flow resistance, which is mainly determined by the shape and size of the flow holes. The formula for the square flow holes is:

(2) $$R_{h}=\frac{1}{N_{f}}\frac{12\mu L_{a}}{L_{f}^{4}}$$

where $N_{f}$ is the numbers of flow holes, μ is the viscosity of the electrolyte, $L_{a}$ is the length of the anode, and $L_{f}$ is the side length of the square flow hole.

From Formulas (1) and (2), it can be seen that for the mechanical module, when the shell of the device remains unchanged, the impact of the structure on the performance of the device is mainly realized by changing the channel flow resistance $R_{h}$. More precisely, it is $N_{f}$, $L_{a}$ and $L_{f}$.

The electrochemical module takes the cathode current as the research object and studies the conversion process from the electrolyte flow velocity $v_{i}$ to the cathode output current $I_{o}$ in the channel. The transfer function of the electrochemical module is:

(3) $$\left|W_{2}\left(\omega \right)\right|=\left|\frac{I_{o}\left(\omega \right)}{v_{i}\left(\omega \right)}\right|=\frac{C}{\sqrt{1+{\left(\frac{\omega }{\omega_{D}}\right)}^{2}}}$$

where C is the conversion constant of the seismometer, $\omega_{D}$ is the diffusion frequency, and the cathode output current $I_{o}$ is:

(4) $$I_{o}=I_{c2}-I_{c1}=Dq\left(\oint^{\;}\left(\nabla c,\vec{n}\right)\right)dS_{c2}-Dq\left(\oint^{\;}\left(\nabla c,\vec{n}\right)\right)dS_{c1}\;I_{o}=I_{c2}-I_{c1}=Dq\left(\oint^{\;}\left(\nabla c,\vec{n}\right)\right)dS_{c2}-Dq\left(\oint^{\;}\left(\nabla c,\vec{n}\right)\right)dS_{c1}$$

where D is the diffusion coefficient, c is the concentration of $I_{3}^{-}$, q is the amount of charge transferred on the surface of the cathode in the electrochemical reaction, $\vec{n}$ is the unit vector perpendicular to the surface of the electrode, and S is the effective area of the cathode.

From Formulas (3) and (4), it can be seen that for the electrochemical module, when the shell of the device remains unchanged, the impact of the structure on the performance of the device is mainly achieved by changing the effective area S of the cathodes. S is a factor that needs to be analyzed.

### 2.2. Numerical Simulation

**Figure 2:**
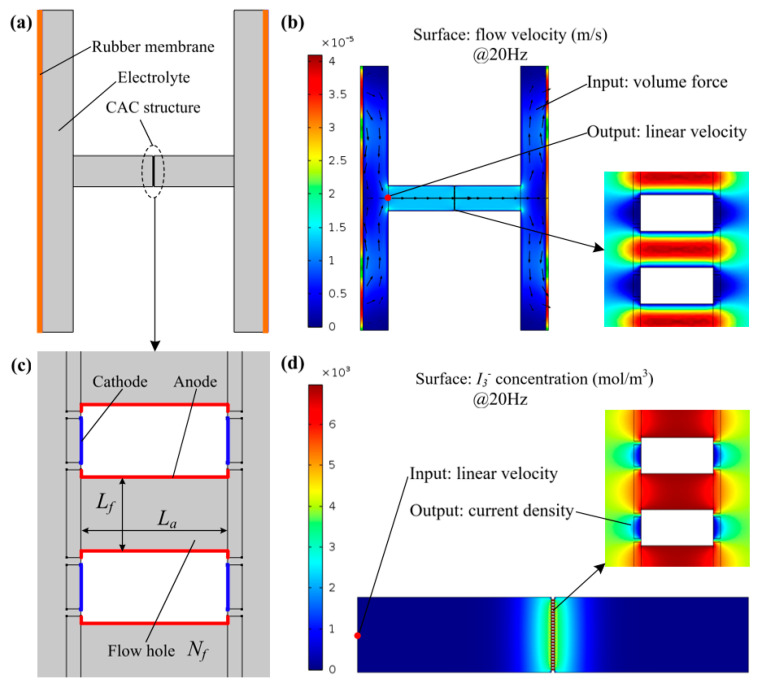
Schematic and preliminary results of the numerical simulations of the mechanical module (**a**) and (**b**) made of key components of the rubber membrane in orange, the electrolyte in gray, and the CAC integrated three-electrode structure positioned within the middle of the flow channel and the electrochemical module (**c**,**d**) made of electrodes and flow holes with the key geometrical parameters of the length of the anode ($L_{a}$), the side length of the square flow hole ($L_{f}$), and the number of flow holes ($N_{f}$).

According to the working principle of the MEMS-based electrochemical seismometer, a finite element simulation was sequentially conducted to model the mechanical and electrochemical modules since it is a complex process. Figure 2a shows the schematic of numerical simulations of the mechanical module made of key components of the rubber membrane in orange, the electrolyte in gray, and the CAC integrated three-electrode structure positioned within the middle of the flow channel. In this simulation, the “Fluid–Solid Interaction” physical field was used where the volume force of the electrolyte was used as an inputting parameter, while linear velocities (parallel to channel walls) around the CAC integrated three-electrode structure were obtained as the outputs of the simulation (see Figure 2b). Figure 2c shows the schematic of the numerical simulations of the electrochemical module made of key components of cathodes, anodes, and flow holes with the key geometrical parameters of the length of the anode ($L_{a}$), the side length of the square flow hole ($L_{f}$), and the numbers of flow holes ($N_{f}$). In this simulation, the “Laminar Flow” and “Electroanalysis” coupled fields were used where linear velocities (uniform distribution) around the CAC integrated three-electrode structure derived from the previous simulation of the mechanical module were used as inputting parameters, while the local current densities around the cathodes were obtained as the outputs of the simulation (see Figure 2d).

In the simulation, the Continuity equation and the Navier–Stokes equation were used to solve the velocity distribution of the electrolyte in the channel. Faraday’s law, the Butler–Volmer condition, and the Nernst–Planck equation were used to solve the liquid mass transfer and electromechanical conversion process of $I_{3}^{-}$ between anodes and cathodes. The boundary conditions included four main aspects. First, the electrolyte was an incompressible liquid in a laminar flow. Second, the solid wall was non-slip. Third, the flow velocity of the electrolyte was zero at the channel wall. Fourth, the ion transport only considered convection and diffusion.

**Figure 3:**
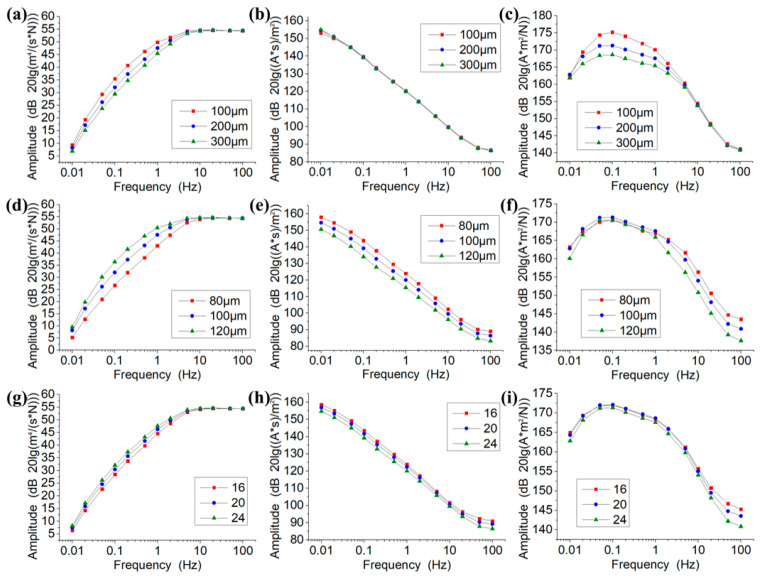
Simulation results of the mechanical module, the electrochemical module, and a combination of the two as a function of the length of the anode (**a**–**c**), the side length of the square flow hole (**d**–**f**), and the number of flow holes (**g**–**i**).

Figure 3a–c shows the simulation results of the mechanical module, the electrochemical module, and a combination of the two as a function of the length of the anode ($L_{a}$, 100–300 μm). As shown in Figure 3a, for the mechanical module, the increase of $L_{a}$ can lead to a decrease in the output within the frequency range of 0.01–10 Hz and has a negligible effect within the frequency range of 10–100 Hz. The reason is that the increase of $L_{a}$ can cause the hydrodynamic resistances of channels to increase. As for the electrochemical module, as shown in Figure 3b, the change of $L_{a}$ has basically no effect on the output since it cannot modulate the areas of cathodes. In combination, the increase of $L_{a}$ can lead to a decrease in the output of the electrochemical seismometer within the frequency range of 0.01–10 Hz and cannot affect its performances within the frequency range of 10–100 Hz (see Figure 3c).

Figure 3d–f shows the simulation results of the mechanical module, the electrochemical module, and a combination of the two as a function of the side length of the square flow hole ($L_{f}$, 80–120 μm). As shown in Figure 3d, for the mechanical module, the increase of $L_{f}$ causes an increase in the output within the frequency range of 0.01–10 Hz, while the curves in the range of 10–100 Hz basically coincide and are not affected by the variation of $L_{f}$. The reason is that the increase of $L_{f}$ can cause the hydrodynamic resistances of channels to decrease. As for the electrochemical module, as shown in Figure 3e, the increase of $L_{f}$ causes the output within the frequency range of 0.01–100 Hz to decrease proportionally, mainly because of the decrease of the areas of cathodes. In combination, the increase of $L_{f}$ can lead to a decrease in the output of the electrochemical seismometer within the frequency range of 1–100 Hz and cannot affect its performances within the frequency range of 0.01–1 Hz (see Figure 3f).

Figure 3g–i shows the simulation results of the mechanical module, the electrochemical module, and a combination of the two as a function of the number of flow holes ($N_{f}$, 16–24). As shown in Figure 3g,h, the influences of $N_{f}$ on the mechanical and electrochemical modules demonstrate a similar trend with a smaller effect in comparison with that of $L_{f}$. In combination, the increase of $N_{f}$ can lead to a decrease in the output of the electrochemical seismometer within the frequency range of 10–100 Hz and cannot affect its performances within the frequency range of 0.01–10 Hz (see Figure 3i).

Based on the results of the numerical simulations and actual considerations of microfabrication, the key geometrical parameters of the CAC integrated three-electrode structure including $L_{a}$, $L_{f}$, and $N_{f}$ were determined to be 200 μm, 90 μm, and 24, respectively.

**Figure 4:**
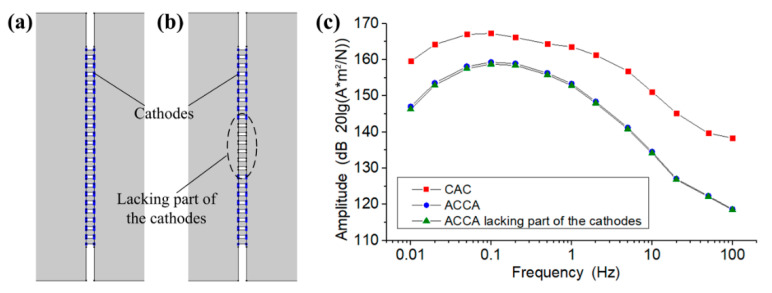
(**a**) The normal structure of the anode–cathode–cathode–anode (ACCA). (**b**) The ACCA structure lacking some of the cathodes. (**c**) The results of the comparison simulation.

The integrated three-electrode structure (labeled as CAC) and the most updated report of the integrated four-electrode structure [22] (labeled as ACCA) were simulated and compared. Among them, the CAC used optimized parameters, while the ACCA was divided into two cases: the normal structure (see Figure 4a) and the structure lacking part of the cathodes (see Figure 4b). Figure 4c shows the results of the comparison simulation. However, two-dimensional simulation can only provide a qualitative evaluation of structural performance, not a quantitative evaluation. Compared with the ACCA, the sensitivity of the CAC has a certain improvement, indicating that the CAC structure has more advantages than the ACCA structure. The reason is that the CAC adopts a differential structure, which can ensure that the electrode reaction is more adequate and rapid. Compared with the ACCA, the sensitivity of the “ACCA lacking part of the cathodes” is almost the same. This indicates that the $I_{3}^{-}$ ions generated from the outer anode are not transported to the cathodes in the middle area for reaction, and some of the cathodes in the middle area are invalid.

## 3. Fabrication

**Figure 5:**
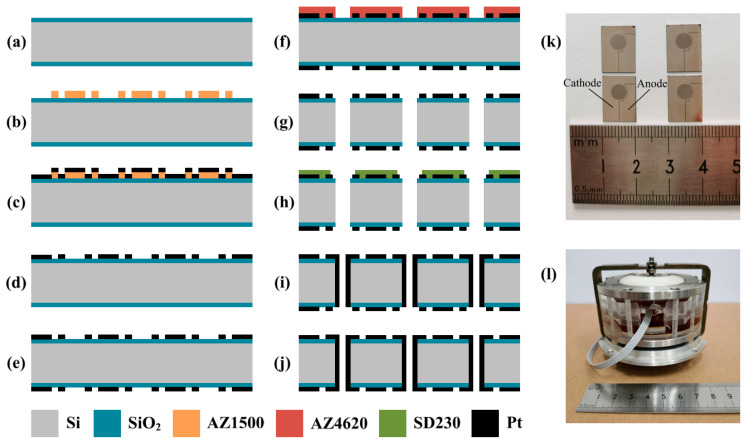
(**a**–**j**) Fabrication process of the CAC integrated three-electrode structure. (**k**) Four prototypes of the CAC integrated three-electrode structures. (**l**) Assembled seismometer.

Figure 5a–j shows the microfabrication process of the CAC integrated three-electrode structure, mainly including thermal oxidization, photolithography, evaporation, sputtering, and etching. In order to maximize the cathode area and improve the sensitivity of the seismometer, the insulation interval between the anode and cathode on the structure surface is 10 μm. Since position deviation has little effect on the performance of the sensor, deviations less than 5 μm were allowed.

A silicon wafer was boiled in concentrated sulfuric acid and deionized water successively to remove contaminated impurities. Then, a silicon dioxide was formed on both surfaces of the silicon wafer by the process of dry oxygen–wet oxygen–dry oxygen (600 nm thick SiO_2_) (a). The front surface of the silicon wafer was spin-coated with photoresist, exposed, and developed (photoresist: AZ1500, developer: 0.6% NaOH) (b). Electrodes were evaporated on the front side of the silicon wafer based on electron beam evaporation (60 nm/250 nm thick Ti/Pt) (c), followed by the lift-off process to form cathodes (d). The back side of the silicon wafer was patterned with electrodes, in alignment with the front surface (e). The front surface of the silicon wafer was spin-coated with thick photoresist, exposed, and developed to form the etch mask (photoresist: AZ4620, developer: 1% NaOH) (f). Reactive ion etching and deep reactive ion etching were sequentially used to remove the layer of silicon dioxide and etch through the silicon wafer (g). The front surface of the silicon wafer with patterned electrodes was attached with a dry film, which was then exposed and developed (dry film: SD230, developer: 0.85% Na_2_CO_3_) (h). Sputtering was conducted to form the sidewall electrodes as anodes (60 nm/250 nm thick Ti/Pt) (i). The back surface of the silicon wafer was again covered by the dry film and sputtered with electrodes to balance the geometry (j).

Figure 5k shows the prototypes of the CAC integrated three-electrode structures followed by the dicing of the silicon wafer. After the step of microfabrication, the electrode structure was sealed by the plexiglass shell and two rubber membranes, followed by the injection of the electrolyte solution (see Figure 5l). In addition, the concentrations of iodine and potassium iodide in the electrolyte were 0.02 mol/L and 2 mol/L, respectively.

## 4. Device Characterizations

### 4.1. Amplitude–Frequency Response

Without an additional compensation circuit and feedback circuit, the original amplitude–frequency characteristics of the CAC and the CME6011 output through current-to-voltage conversion resistance (both using 1 kΩ) were tested. Sinusoidal voltage signals of different frequencies were generated by a signal generator and amplified by a power amplifier to drive the vibrating table to generate vibration signals. The CAC and the CME6011 were placed on a low-frequency vibration table to test the output voltage at each frequency point and the sensitivity curves were drawn in combination with the input velocity.

**Figure 6:**
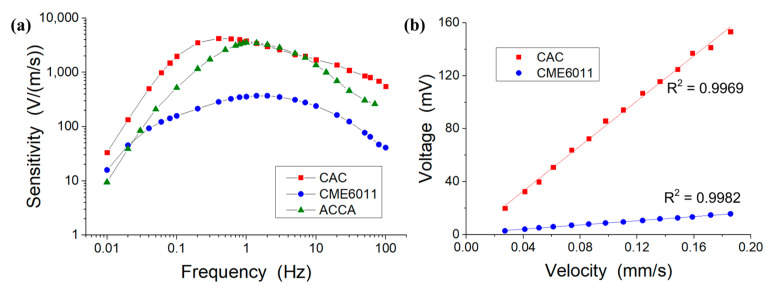
(**a**) Amplitude–frequency responses of the MEMS-based electrochemical seismometer relying on a CAC integrated three-electrode structure (CAC), the commercially available electrochemical seismometer based on woven metallic meshes (CME6011), and the most updated report of the MEMS-based electrochemical seismometer relying on the integrated four-electrode structure (ACCA) [22]. (**b**) Linearity comparison between the CAC and the CME6011.

Figure 6a shows the amplitude–frequency responses of the MEMS-based electrochemical seismometer relying on a CAC integrated three-electrode structure, where the device sensitivity was shown to first increase with the characterization frequency, peak at the mid-frequency domain, and decrease with the further increase of the characterization frequency. In the low-frequency domain, the increase of the device sensitivity as a function of inputting frequency was due to the fact that the mechanical module, as the high-pass link, played the main role. In the high-frequency domain, the decrease of the device sensitivity as a function of inputting frequency was due to the fact that the electrochemical module, as the low-pass link, played the main role.

In addition, key parameters of the developed MEMS-based electrochemical seismometer relying on a CAC integrated three-electrode structure, including maximal sensitivity and 3d bandwidth, were quantified as 4246.20 V/(m/s) @0.4 Hz and 0.16–2.01 Hz, respectively (see Table 1). When these parameters were compared with the CME6011, the commercially available electrochemical seismometer relying on woven metallic meshes, the sensitivity of the CAC was an order of magnitude higher than that of the CME6011. As to the bandwidth, the seismometer developed in this study performed better in the low-frequency domain in comparison with the CME6011 (0.16 Hz vs. 0.33 Hz), although the CME6011 had a larger bandwidth range (see Figure 6a). In terms of linearity, in the velocity range of 0.025–0.185 mm/s, the correlation coefficients of the fitted curves of the CAC and the CME6011 were 0.9969 and 0.9982, respectively. This showed that the linearity of the two was basically the same (see Figure 6b).

Furthermore, the MEMS-based electrochemical seismometer relying on a CAC integrated three-electrode structure was also compared with the most updated report of the MEMS-based electrochemical seismometer relying on the integrated four-electrode structure [22] (labeled as ACCA, see Figure 6a). Compared with the ACCA, the sensitivity of the CAC was higher, especially in the low-frequency domain of 0.01–1 Hz and the high-frequency domain of 10–100 Hz. When the working bandwidth was compared, once again, the seismometer developed in this study performed better in the low-frequency domain in comparison to the ACCA (0.16 Hz vs. 0.48 Hz), which was thus more suitable for the monitoring of low-frequency vibrations (see Figure 6a).

From the perspective of electrode structure, the ACCA adopted the structure with a large ring and a small circle. The outer anode was far away from the cathode in the center of the structure, so it was difficult for $I_{3}^{-}$ ions to reach the central area. The CAC adopted the differential structure, and the $I_{3}^{-}$ ions needed for the cathode reaction were all provided by the anode on the sidewall of the nearest flow hole, which can ensure a sufficient reaction. When the vibration detected by the seismometer was a high-frequency signal, the electrolyte produced a high-frequency vibration in a small area. For the ACCA structure, it was difficult for the $I_{3}^{-}$ ions generated by the outer anode reaction to move to the cathode in the central area to react, resulting in a loss of high-frequency sensitivity. For the CAC structure, the $I_{3}^{-}$ ions generated at the anode of each flow hole can quickly reach the nearby cathode to react, and the high-frequency sensitivity was improved. When the vibration detected by the seismometer was a low-frequency signal, the electrolyte produced a wide range of low-frequency flow. For the ACCA structure, most of the $I_{3}^{-}$ ions generated by the outer anode reaction were consumed by the outer cathode reaction in the process of moving to the center. The $I_{3}^{-}$ ions reaching the cathode in the central area were also very small, and the low-frequency sensitivity was affected. As for the CAC structure, the distance between the anode and the cathode was very small, and the large-scale movement of the electrolyte did not cause an insufficient supply of $I_{3}^{-}$ ions around some cathodes. Thus, the low-frequency sensitivity can also be high.

### 4.2. Noise Level

In the absence of vibration signals, the voltage noise signal was collected for a duration of 600 s, and the Fourier transform was used to convert the data of the time domain into the frequency domain. Combined with the sensitivity curve that had been obtained, the voltage noise signal at each frequency point was divided by the corresponding sensitivity to obtain the power spectrum of velocity noise.

**Figure 7:**
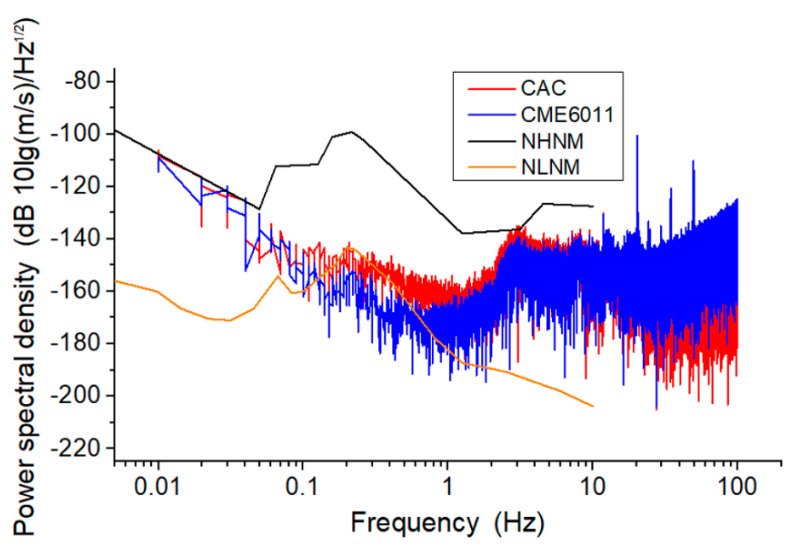
Noise level of the MEMS-based electrochemical seismometer relying on a CAC integrated three-electrode structure (CAC) and the commercially available electrochemical seismometer based on woven metallic meshes (CME6011). NHNM, new high-noise model of the earth; NLNM, new low-noise model of the earth.

Figure 7 shows the noise level of the MEMS-based electrochemical seismometer relying on a CAC integrated three-electrode structure. The noise intensity was shown to decrease initially with the increase of the frequency (0.01–1 Hz), because the 1/f noise played a major role in this frequency domain. Additionally, there was no clear trend between the frequency and noise intensity of the frequency (1–100 Hz), because the relationship between the thermal noise, the convection noise, and other factors were complicated in this frequency domain.

When the noise levels of the CAC and the CME6011 were compared for the frequency domains of 0.01–0.1 Hz and 1–10 Hz, comparable noise levels of the CAC and the CME6011 were observed. For the frequency domain of 0.1–1 Hz, the noise level of the CAC was slightly higher than that of the CME6011, while in the frequency domain of 10–100 Hz, the noise level of the CAC was slightly lower than that of the CME6011. More specifically, quantitative comparisons of noise levels between the CAC and the CME6011 were as follows: −108.28 dB vs. −108.89 dB at 0.01 Hz, −175.84 dB vs. −178.34 dB at 1 Hz, and −145.64 dB vs. −128.02 dB at 100 Hz.

### 4.3. Response of Random Vibrations

**Figure 8:**
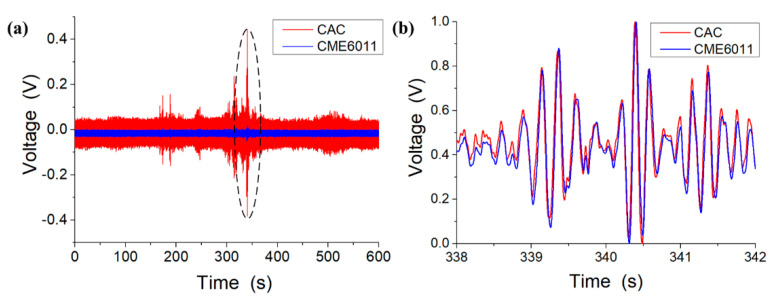
The preliminary (**a**) and normalized (**b**) responses of random vibrations of the MEMS-based electrochemical seismometer relying on a CAC integrated three-electrode structure (CAC) and the commercially available electrochemical seismometer based on woven metallic meshes (CME6011).

Figure 8 shows the responses of the random vibrations of the MEMS-based electrochemical seismometer relying on a CAC integrated three-electrode structure (CAC) and the commercially available electrochemical seismometer based on woven metallic meshes (CME6011). These preliminary results (see Figure 8a) show that the CAC produced higher voltages than the CME6011 under the same condition of vibration since the sensitivity of the CAC was higher than that of the CME6011. After output normalization, comparable results were obtained between the CAC and the CME6011 with and without random vibrations (see Figure 8b). For instance, for the random vibration between 338 and 342 s, a high correlation coefficient between the CAC and the CME6011 (0.985) was obtained, which further validated the performance of the MEMS-based electrochemical seismometer relying on a CAC integrated three-electrode structure.

## 5. Conclusions

This study developed a CAC integrated three-electrode electrochemical seismometer where key geometrical parameters of the CAC structure were optimized based on numerical simulations. MEMS processes, including the key steps of photolithography, evaporation, sputtering, and etching, were developed for the fabrication of the seismometer. The key performing parameters of the CAC seismometer, including sensitivity and noise level, were characterized, producing an improvement of an order of magnitude and a comparable level, respectively, compared with the CME6011 commercial counterpart. Compared with the most updated report of the MEMS-based integrated four-electrode seismometer [22], the CAC-based microseismometer developed in this study features with a simplified fabrication process, a comparable sensitivity, and a better performance in the low-frequency domain. Due to the aforementioned results, the MEMS-based electrochemical seismometer relying on a CAC integrated three-electrode structure may provide a new perspective in seismic observations and resource explorations.

## Figures and Tables

**Table 1:** Performance Comparisons of Three Representative Electrochemical Seismometers.

Performance	CAC	CME6011	ACCA
Maximum sensitivity	4246.20 V/(m/s) @0.4 Hz	371.15 V/(m/s) @1.4 Hz	3555.57 V/(m/s) @1 Hz
3 dB working bandwidth	0.16–2.01 Hz (1.06 decades)	0.33–8.09 Hz (1.37 decades)	0.48–3.98 Hz (0.86 decades)
